# Heavy Physical Work: Cardiovascular Load in Male Construction Workers

**DOI:** 10.3390/ijerph13040356

**Published:** 2016-03-23

**Authors:** Lars-Kristian Lunde, Markus Koch, Kaj Bo Veiersted, Gunn-Helen Moen, Morten Wærsted, Stein Knardahl

**Affiliations:** 1 National Institute of Occupational Health, Gydas vei 8, 0336 Oslo, Norway; markus.koch@stami.no (M.K.); bo.veiersted@stami.no (K.B.V.); morten.waersted@stami.no (M.W.); stein.knardahl@stami.no (S.K.); 2 Oslo University Hospital, Trondheimsveien 235, 0514 Oslo, Norway; g.h.o.moen@studmed.uio.no

**Keywords:** construction work, general health, musculoskeletal pain, physical demands, work ability

## Abstract

This study aimed to elucidate cardiovascular loads (CVL) in construction workers during work and leisure by relative heart rate (RHR) over several days. Furthermore, we sought to evaluate the level of CVL in relation to individual factors, work ability, musculoskeletal pain and subjective general health. From a group of 255 construction workers responding to the baseline questionnaire, the CVL during work and leisure time was determined by recording RHR in 42 workers over 3–4 days. Almost 60% of the workday was spent below 20% RHR. The mean RHR during work for all participants was 16% RHR, with large differences between professions. On average, the 42 workers spent 14% of the workday at a RHR above 33%, and four subjects (10%) had a mean RHR above 33% during work. Eight (19%) of the participants had a mean length of their workday exceeding calculated maximal acceptable work time. Seven persons (17%) experienced on average one or more episode(s) of 5 min or more continuously above 33% RHR. The cardiovascular load at work was significantly associated with age and $\dot{V}$O_2max_, but not with work ability, musculoskeletal pain or subjective general health.

## 1. Introduction

Physical demands at work are considered an important risk factor of several musculoskeletal disorders (MSD; see [1] for a systematic review). Moreover, heavy physical work may be associated with level of work ability [2]. Heavy physical work is a general term, encompassing both working at high levels of aerobic load relative to maximum oxygen uptake, handling of heavy objects, and performing tasks demanding sustained exertion at high levels of force. Hence, the term heavy physical work does not specify pathogenic factors to target for workplace interventions to prevent health problems.

Individual performance and production output depend on being able to sustain workload over a period of time, which depends on both individual capacity and type of tasks performed. Several authors have suggested guidelines for work-intensity and -duration to ensure safety, health, and productivity of employees [3,4]. Åstrand and coworkers have pointed out that the physical workload should be determined with indirect calorimetry and that the oxygen uptake must be evaluated with regard to the capacity of the working muscles [5]. A criterion for the limit of acceptable workload is the occurrence of a marked increase in heart rate (HR; >10 beats per minute) after a period of working at a steady state with constant HR [6], a sign of physiological fatigue. Rodgers and co-workers proposed that a workload of one third of the individual’s maximum capacity should be the upper limit for an eight-hour workday [7]. Others have described workload limits for physically demanding jobs through “maximal acceptable work time” (MAWT), a term referring to workloads that can be sustained by an individual in physiologically steady state without causing exhaustion or discomfort [8]. 

Work in the construction industry is generally considered physically demanding and construction workers show high prevalence of musculoskeletal pain [9,10]. Higher rates of disability, lowered physical function, and reduction in muscular strength have been found in occupations with high levels of physical demands [11,12,13]. However, the majorities of studies of heavy physical work were based on subjective measurements of physical demands. The validity and reliability of these exposure measurements are questionable [14]. Van der Molen and co-workers did measure cardiovascular demands objectively by HR and oxygen consumption in groups of construction workers during several work tasks [15,16,17]. These studies had a limited number of participants (*N*= 8, 10, and 15, respectively) and measured demands during one single period. Two recent studies have objectively measured cardiovascular load for more than one day within other occupations commonly considered as physically demanding, female hospital cleaners [18] and an unspecified group of blue-collar workers [19]. Still, within construction work there is a paucity of studies with objective measurements of cardiovascular load over several days. Therefore, there is a need for knowledge of physical workload based on objective measurement of cardiovascular load in employees performing heavy physical work in the construction sector.

It seems a paradox, that physical activity during leisure time is considered health-promoting and essential for maintaining and increasing physical capacity and work ability [20,21,22], while physical demands at work may be harmful. One might expect that heavy physical work would produce positive training effects [23,24]. However, negative or no training effects from a life-time of heavy work exposure have been reported [25,26]. Differences in patterns of physical activity during work and leisure could be an important factor when explaining this phenomenon [27]. Moreover, heavy physical work may be a risk factor for leisure time physical inactivity [28]. Therefore, information on physical demands and activity patterns during both work and leisure is needed. 

There is a linear relationship between HR and oxygen consumption during a bout of work or exercise [29]. Hence, HR may be measured as a proxy of workload or work intensity or aerobic strain. Some previous recommendations of workload have related work duration to workload operationalized as % of maximum O_2_ uptake ($\dot{V}$O_2max_) or % of maximum HR (HR_max_) for the individual. HR_max_ depends on age and there are several formulas for calculating HR_max_ [30]. One problem with the %HR_max_ approach is the fact that resting HR (HR_min_) never is zero, hence the percentage of HR_max_ does not represent load above the resting state. Furthermore, some physically fit individuals exhibit very low HR_min_, hence their range of HR-variation (HR reserve) is larger for a given age. The relative HR (RHR or % of HR reserve) takes HR_min_ into account by subtracting HR_min_ from the HR measured during work and from HR_max_. 

In the present study we aimed to elucidate cardiovascular loads in construction workers during work and leisure by relative HR (RHR) from objective measures over several days. We further evaluated the level of cardiovascular load in relation to individual factors, work ability, MSDs and general health.

## 2. Methods

### 2.1. Participants

Subjects for this study were recruited from three large construction enterprises during April–September 2014. A total of 579 employees were invited to fill out a baseline questionnaire and give their consent to participate. Two hundred and fifty-five answered the baseline questionnaire and 161 stated they were also willing to participate in ambulatory technical measurements. From the 161 a sample of 57 was invited to the technical recordings presented in this paper. The 57 were selected to best fit logistics (based on availability and work schedules) and to give a reasonable representation of occupational titles. The construction sector consists of a high proportion of men, hence all participating volunteers investigated by technical measurements in this study are men. Individual characteristics for participants in the technical measurement group and the questionnaire group are shown in Table 1. Exclusion criterion for answering the questionnaire was inadequate skills in reading and writing Norwegian. Diagnosed cardiovascular disease or known allergic reaction to plaster/tape/bandages were exclusion criteria for technical measurements of HR. Subjects with considerable musculoskeletal pain on the test day or diagnosed with back or shoulder disorders, were not subjected to physical capacity tests they were unable to perform. 

### 2.2. Compliance with Ethical Standards

All participants were informed of the purpose and content of the study and signed an informed consent prior to participation. The study was conducted in accordance with the 1964 Helsinki declaration and approved by The Regional Committee for Medical and Health Research Ethics in Norway (2014/138/REK south-east D).

### 2.3. Study Procedure

Participants volunteering for the study answered first the baseline questionnaire before proceeding to a physical examination (including weight and height measurements) carried out by a physician or nurse. If none of the exclusion criteria were present, the participant carried out a physical fitness test determining aerobic fitness and muscular strength. On a succeeding (separate) workday morning, instruments for ambulatory technical measurement were attached. The instruments for recording physical exposure sampled 24-h a day for three to four consecutive days or until deliberately removed by the subject. Measurement were targeted to include at least two working days. On the first day of measurement all participants were given a small diary where they should note time of day they got out of bed in the morning, started work (if workday), ended work (if workday), and went to bed at night for the days they were measured. Additionally, if the instruments at any time were detached subjects were instructed to note time periods when the monitor was not worn. They were also given extra electrodes and instruction on how to attach the measurement equipment if it detached unintentionally. 

### 2.4. Questionnaire

The following self-reported measures from questionnaire were included in this study: seniority, height, weight, smoking status, perceived exertion at work [31], work ability [32], perceived health [33], musculoskeletal disorders previous four weeks [34] and level of leisure time physical activity [35]. 

#### 2.4.1. Smoking

Smoking status was determined by the question: “do you smoke or have you ever smoked?” with the four response alternatives: No, never (0), yes, but not anymore (1), yes, occasionally (2) and yes, every day (3). The responses were dichotomized in to non-smoking (0–1) and smoking (2–3). 

#### 2.4.2. Perceived Exhaustion during Work

The question “how physically demanding do you normally find your work?” with a response scale of 13 categories, ranging from “not exhausting at all” to “maximally exhausting” measured self-reported exertion during work [31]. 

#### 2.4.3. Work Ability 

Work ability was measured by a single item taken from the Work Ability Index: “current work ability compared with lifetime best”. The score range for this question is from 0 (“completely unable to work”) to 10 (“work ability at its best”). This single item has previously shown strong predictive value for health outcomes [32]. 

#### 2.4.4. Perceived Health

Self-perceived health was measured using the question: “How is your general health at present?”. Participants had five response alternatives ranging from excellent to poor [33]. 

#### 2.4.5. Musculoskeletal Disorders

Musculoskeletal pain in neck, shoulders, back (upper, lower), elbow, hip, knee and foot/ankle were measured by assessing pain intensity and duration during the previous four weeks. Pain intensity was classified by participants to be no pain (0), mild pain (1), moderate pain (2) or severe pain (3) with the pain duration alternatives 1–5 (1), 6–10 (2), 11–14 (3) and 15–28 (4) days. From these answers a pain score was calculated, ranging from 0 (no pain × no duration) to 12 (severe pain × 15–28 days). A musculoskeletal complaint-severity index (MSI) was computed as a mean of the pain scores for all pain sites [34]. 

#### 2.4.6. Self-Reported Leisure-Time Physical Activity Level

Leisure-time physical activity level was determined by a single item. The participants reported which of the following activities levels that corresponded best to their own level the previous four weeks: (1) Almost completely inactive (e.g., reading, watching TV, movies); (2) Some physical activity at least four hours per week (e.g., bicycling, walking, gardening); (3) Regular activity (e.g., running, tennis); (4) Regular hard physical training for competition several times per week [35].

### 2.5. Physical Capacity Assessment

#### 2.5.1. Aerobic Fitness 

Aerobic fitness was established using a standardized cycle ergometer test (Ergometer 839 E, Varberg, Sweden) [36]. Based on assumed state of fitness an external power was set between 75 and 150 watts and subjects performed a cycling rate of 50 revolutions per minute. The test was terminated when heart rate obtained a steady state at a level greater than 120 beats per minute (bpm), normally within the period between the 5th and 6th minute. The mean steady-state heart rate was used to estimate $\dot{V}$O_2max_ based on the Åstrand nomogram [37]. 

#### 2.5.2. Muscular Strength 

Handgrip strength was tested according to standardized procedures [38] by a hand dynamometer (Lafayette Instrument, Lafayette, IN, USA). For each hand, the highest obtained value of two attempts was used. 

### 2.6. Assessment of Cardiovascular Load

#### 2.6.1. Instrumentation

Heart rate recording was carried out with, Actiheart 4 (Camntech, Cambridge, UK), a small chest-worn monitoring device consisting of two clips attached to standard electrocardiogram electrodes (Blue sensor VL-00-S/25 Ambu, Ballerup, Denmark) placed at the apex of the sternum and at the left intercostals at the level of the 6th and 7th costae [39]. Before affixing the electrodes, the skin was prepared by shaving and cleaning with ethanol spirits. Analog signals of the Actiheart were filtered (10 Hz–35 Hz) and sampled with a frequency of 128 Hz. The Actiheart measures HR by calculating the R-R intervals of the ECG. For analysis of HR a custom made software, Acti4 (National Research Centre for the Working Environment, Copenhagen, Denmark and Federal Institute of Occupational Safety and Health, Berlin, Germany) was used [19]. The Actiheart produces reliable 24-h measurement in physically active workers [18].

#### 2.6.2. Data Processing

Based on the diary data, each day was categorized into periods: before work, work, after work, sleep and leisure (off days). In this study the periods analyzed were work time and leisure time on work days (before and after work). Sleep periods and leisure on off days are not reported here. To be eligible in the analysis participants needed to have valid measurement periods (work or leisure) lasting four hours or longer than ≥75% of the length of a normal period. The definition of a normal period was the average of the measured periods. Data were also excluded if beat error exceeded 50% for a measurement period, defined as HR <35 or >230 bpm or >15% difference between two succeeding beats. In addition, all measurement periods were visually checked. Data for the valid time periods within work and leisure categories were then aggregated and averaged for each individual. 

To evaluate the relative cardiovascular load the RHR during work and leisure respectively, was calculated as follows:

$$RHRwork=\frac{\left(\text{HRwork}-\text{HRmin}\right)}{\left(\text{HRmax}-\text{HRmin}\right)}\times 100\;\text{and}\;RHRleisure=\frac{\left(\text{HRleisure}-\text{HRmin}\right)}{\left(\text{HRmax}-\text{HRmin}\right)}\times 100$$

In this equation the HR_max_ is given by 208 – 0.7 × age [30]. The HR_min_ entered into the equation was a sex- and age-adjusted value obtained from a Norwegian population study (HUNT3) [40] (20–29 yrs; 69.4 bpm, 30–39 yrs; 68.7 bpm, 40–49 yrs; 68.2 bpm, 50–59 yrs; 68.6 bpm, 60–69 yrs; 68.1 bpm). HR_work_ and HR_leisure_ in the equation is the mean HR measured for the respective periods. 

To calculate MAWT the equation 26.12 × e^−4.81 × RHR/100^ using mean RHR during work was implemented [8]. 

### 2.7. Statistical Analysis

Normal distribution of independent variables was tested by the Shapiro-Wilk tests of normality. Differences between questionnaire and technical measurement groups and differences in percentage distribution of RHR ranges between work and leisure were tested with independent samples *T*-tests and Mann-Whitney U tests. The associations between individual factors, work ability, and MSD and CVL at work were tested with simple and multiple linear regression analyses. Associations between independent variables were assessed by Pearson correlation prior to multiple regression. If variables were highly correlated (r > 0.6), the variable with highest predictive value was used in the analysis. For the statistical analyses IBM SPSS Statistics 23 (IBM Corporation, Armonk, NY, USA) was used. Significance level was set at *p* = 0.05.

## 3. Results 

There were significant (*p* < 0.05) differences in smoking status and gender distribution between the questionnaire group and the technical measurement group, with more men and smokers in the latter (Table 1). The technical measurement group did not differ from the questionnaire group in: age, height, body mass, body mass index, number of normal working hours, perceived exhaustion at work, perceived health or work ability. 

**Figure 1:**
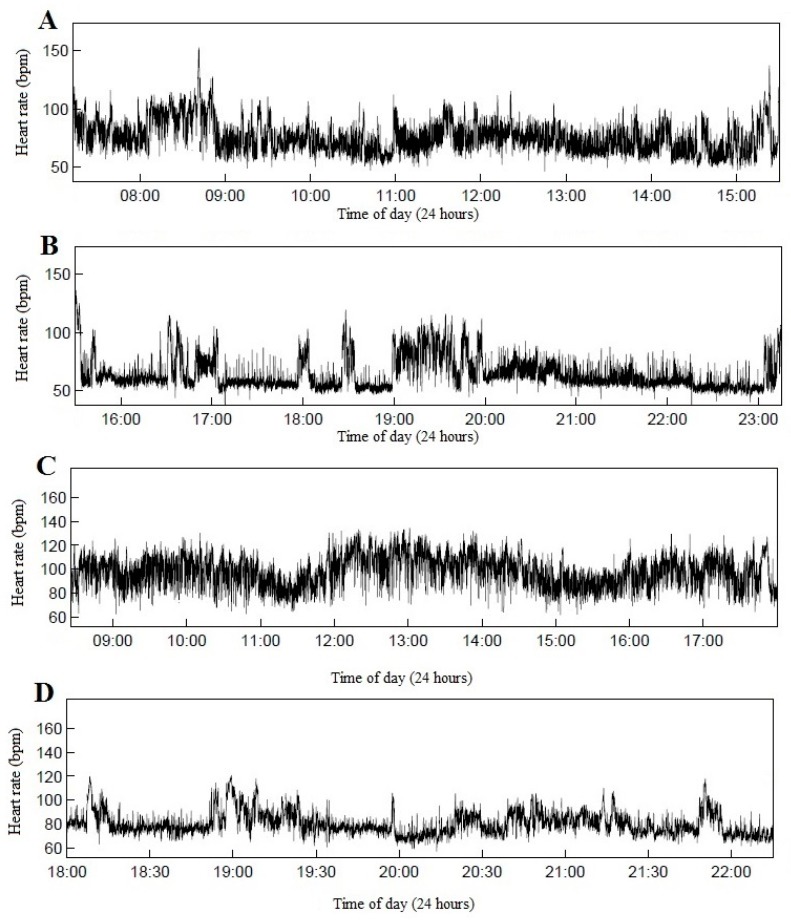
Examples of single periods of measured work and leisure heart rate for a foreman and a carpenter. (**A**) work foreman; (**B**) leisure foreman; (**C**) work carpenter and (**D**) leisure carpenter.

Of the 57 who recorded HR, 15 subjects were not included in the final analysis due to technical measurement error (blank measurements), too many measurement periods with beat error above 50% or unfulfilled length of measurements criteria. Hence, the sample available for analysis of cardiovascular strain at work was 42. Two subjects did not have any valid measurements outside working hours, thus 40 subjects were available for leisure time HR analysis. A total of 85 days of work were measured with a mean length of measured workday being 8.1 h (±2.2). For leisure time measurements the total was 81 days with a mean length 7.3 h (±2.6). Figure 1 shows example measurements taken from a foreman and a carpenter.

### 3.1. Cardiovascular Load during Work

The average RHR during work for all participants was 16.4% (±11.4). The distribution within different ranges of RHR showed that for 58.6% (±28.5) of the work day RHR levels were lower than 20% RHR. Furthermore, for 19.5% (±13.3) and 11.1% (±10.2) of the day RHR level ranged between 20%–29% RHR and 30%–39% RHR, respectively. A small proportion of the day, 5.0 percent (±7.4) of the day in a RHR between 40%–49% and 1.9% (±3.6), the work was accompanied by cardiovascular demands in range of 50%–59% RHR. 

**Figure 2:**
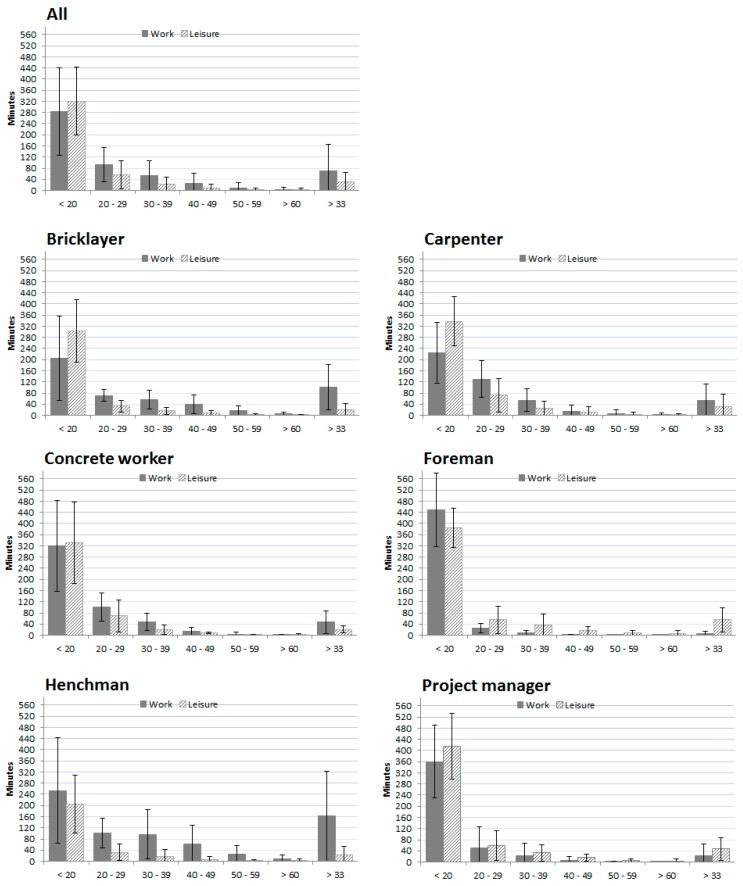
Relative heart rate distribution during work and leisure presented as minutes with standard deviation. *During work*: All; *n* = 42, Bricklayer; *n* = 5, Carpenter; *n* = 12, Concrete worker; *n* = 8, Foreman; *n* = 4, Henchman; *n* = 8, Project manager; *n* = 5. *During leisure*: All; *n* = 36, Bricklayer; *n* = 3, Carpenter; *n* = 11, Concrete worker; *n* = 7, Foreman; *n* = 4, Henchman; *n* = 6, Project manager; *n* = 5.

**Figure 3:**
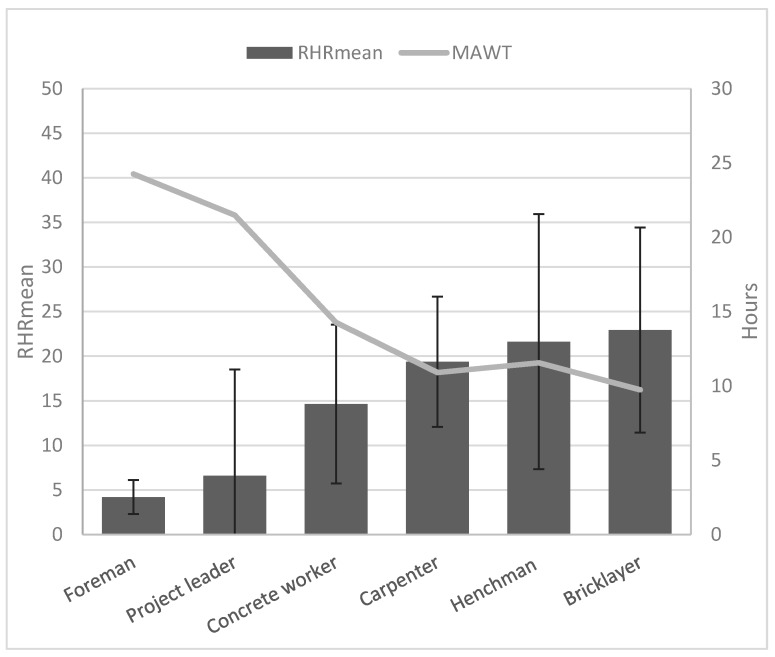
Mean relative heart rate during work and maximal acceptable worktime for professions.

For the group as a whole, 14.4% (±18.4) of the working day was spent above 33% RHR. Out of 42 subjects, 10% (4) did have a mean RHR above 33% during work, 90% (38) did not. Foremen and project leaders spent fewest minutes above 33% RHR during work (6.5 ± 8.3 min and 22.9 ± 42.4 min), see Figure 2. Foremen and project leaders had lowest mean RHR (4.2% ± 2.0% and 6.6% ± 11.9%) while carpenters, henchmen and bricklayers all had a mean RHR of approximately 20% (19.4 ± 7.2, 21.6 ± 14.3 and 22.9 ± 11.5), see Figure 3. 

#### Maximal Acceptable Work Time and Continuous Work and Rest Periods

The average MAWT for this sample was 14.1 (±8.4) hours, while mean length of work shifts was 8.1 (±2.2) hours. Eight (19%) subjects exhibited work lengths exceeding mean calculated MAWT (1 out of 9 with mostly administrative tasks and 7 out of 33 with manual tasks), 34 (81%) did not. Foremen had highest and bricklayers had the lowest MAWT, see Figure 3. 

**Figure 4:**
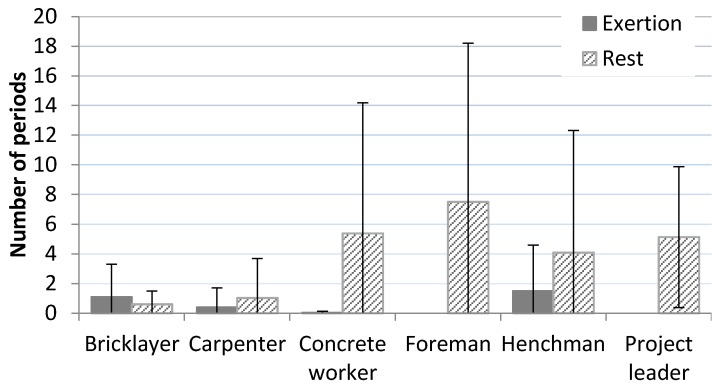
Number of episodes with mean RHR above 33% (exertion) or below 10% (rest) continuously for 5 min or more.

Seven persons had on average one or more episode per day of RHR above 33% continuous for 5 min duration or more (exertion periods). Five persons showed one or more episodes of 10 min or more and one person had episodes of RHR above 33% continuously for 15 min or longer. See Table 2. Henchmen had highest number of continuous periods of more than 5 min above 33% RHR, while there were no such periods among project leaders and foremen. Foremen did have the highest mean number of rest periods, defined as periods of 5 min continuously below 10% RHR, with 7.5 (±10.7), see Figure 4.

### 3.2. Cardiovascular Load during Leisure Time

The mean load during leisure time was significantly lower compared to work, with mean RHR of 9.2% (±7.8), *p* < 0.01. Moreover, when compared to work, the distribution of the leisure time periods spent in different RHR ranges showed that a significantly higher proportion was spent below 20% RHR (75.2% ± 16.9%; *p* < 0.01) and significantly lower proportions in RHR ranges of 20%–29% (19.5% ± 13.3%; *p* < 0.01), 30%–39% (11.1% ± 10.2% ; *p* < 0.01) and 40%–49% (5.0% ± 7.4%; *p* < 0.05), see Figure 2. 

### 3.3. Cardiovascular Load and Individual Factors

#### 3.3.1. Age and Seniority

Unadjusted linear regression analysis showed RHR during work to be significantly associated with age (β = −0.298, *p* < 0.05), indicating decreasing levels of RHR with increasing age. Seniority (years in profession) showed similar tendency, however, did not reach significance criteria (β = −0.234, *p* = 0.085). In the adjusted multiple linear regression model age remained significant (β = −0.414, *p* < 0.01). Seniority was strongly (r = 0.845, *p* < 0.001) correlated to age. 

#### 3.3.2. Aerobic Fitness, Leisure Time Physical Activity and Muscular Strength 

RHR was dependent on aerobic fitness level, showing a significant association to estimated $\dot{V}$O_2max_ (β = −5.924, *p* < 0.01). Higher levels of $\dot{V}$O_2max_ was associated to lower RHR during work. The $\dot{V}$O_2max_ variable remained significant (β = −5.098, *p* < 0.01) in the adjusted analysis. Higher self-reported physical activity levels did also seem to lower RHR during work, but was not significant. Further, we were unable to find any association between RHR and hand strength.

#### 3.3.3. Work Ability 

There was a trend towards lower reported work ability with higher RHR during work. However, this did not reach customary criteria for statistical significance (β = −1.844, *p* = 0.076) and was cancelled out in the adjusted analysis.

#### 3.3.4. Musculoskeletal Pain and Perceived Health

We did not find any association between RHR during work and MSI. Similarly, perceived health did not show any significant associations to RHR. Additionally, there was no associations between leisure time RHR and MSI or perceived health. 

#### 3.3.5. Perceived Exhaustion 

Self-reported perceived exhaustion at work was signficantly associated to levels of RHR. Those reporting high level of perceived exhaustion did also tend to have higher cardiovascular loads during work (β = 1.598, *p* < 0.05). This relationship was cancelled out in the adjusted analysis. 

#### 3.3.6. Smoking 

Our analysis showed no association between smoking and level of RHR during work. See Table 3 for the adjusted and unadjusted linear regressions. 

## 4. Discussion

The results demonstrated cardiovascular load characteristics in male construction workers during work and leisure. Further, the association between RHR at work and the participants’ age and aerobic fitness level is highlighted. We did not find any significant associations between cardiovascular demands at work and work ability, musculoskeletal complaints or general perceived health.

For the group as a whole an average workday was characterized by most time spent in ranges below 20% RHR and less time in higher ranges. For an average workday of 8 h, approximately 5 h were below 20% RHR, while approximately 40 min were spent in ranges above 40% RHR. A limited proportion of the participants (10%) had a mean RHR above the recommended threshold of 33% RHR and for the whole group approximately 70 min were in load ranges above this level of cardiovascular load on an average workday. Carpenters, henchmen and bricklayers represented professions with the highest mean RHR. Compared to carpenters and bricklayers, henchmen did have more episodes of both continuous exertion and rest periods, indicating a somewhat different work pattern. Foremen and project leaders seemed to have less physical demands, with lowest levels of RHR, no continuous periods of exertion and highest number of rest periods. This reflects that different professions within construction will have different physical demands, which should be taken into consideration when evaluating the construction sector. The physical demands of construction supervisors (e.g., foremen and project leaders) have previously been very scarcely investigated [41].

Previous studies investigating masons, bricklayers and plasterboard work (carpenter task) during a full workday have found mean RHR ranging from 21 to as high as 39% RHR, with factors as brick and plasterboard sizes as important load varying factors [15,16,17]. From our data the three professions found to have the highest cardiovascular demands (carpenter, bricklayer and henchman) exhibited HRs in the lower part of this range. However, the above-mentioned studies measured cardiovascular loads for one workday only. We found that the recorded HRs were significantly higher during the first day of measurement compared to following workdays (Koch *et al.* work in progress). This finding indicates that work behavior may be altered the on first day of measurement. Still, compared to the general working life [42], construction workers exhibit a higher level of cardiovascular load. 

The mean MAWT for this sample was 14 hours, and a mismatch between length of workday and MAWT were found in approximately 1 in 5 individuals. A mean RHR of 24.4% would represent MAWT equal to the average workday of 8.1 h. Thus, all professions were within these limits. With this said, the distribution within RHR ranges and exertion/rest periods does imply that construction work is not a physiologically steady-state situation, but is rather fluctuating between levels of cardiovascular load. Therefore, this kind of work may not fulfil the assumptions behind the MAWT-equation, which is set pace ergometer cycling [8]. Additionally, we may expect load carrying tasks to need additional predicting factors [43]. Hence, there is a need for new approaches to estimate workload limits in physical occupations. 

*Age* was significantly associated with RHR during work. Increasing age was associated with reduced cardiovascular load, indicating that younger workers had higher cardiovascular loads during work, compared to older workers. Similarly, Gupta and colleagues found seniority to be significantly more prevalent in workers with low RHR during work, compared to those with high RHR [19]. Possibly, higher seniority workers allocate the more physically demanding tasks to younger workers. Alternatively, inexperienced workers perform tasks at a higher physiological cost than more experienced workers [7]. It is possible that the senior workers are a selection of more fit individuals orthat senior workers are relocated to less physically demanding professions within construction. However, as found in the general population, $\dot{V}$O_2max_ decreased significantly with age in our sample (results not shown), and there were no significant age difference between professions measured.

$\dot{V}$O_2max_ was significantly associated with RHR during work. An increase in aerobic fitness will result in work being less physically demanding, with lower RHR as long as the level of physical demands remains unchanged. Even though there are differences in physical demands between professions, the relative demands for each person will be individually determined by level of fitness. We recorded large individual differences in the relative physical demands within the same profession. 

There is an ongoing discussion concerning the paradoxical effect of physical exercise: seemingly negative effects of high physical activity at work and the health-improving effects of physical activity in leisure time [44,45]. Our study shows that few individuals had continuous periods with RHR above one-third of their capacity and very few minutes were spent above 60% RHR during the workday. Intensive bursts of exhausting physical activity are needed to achieve a training effect on the cardiovascular system [46]. Thus, the combination of duration and intensity seen in construction work do not meet levels required to achieve training effects. For the 10% of our cohort having a mean RHR above one third of maximal capacity, the demands may possibly have a negative effect, rather than a training effect [47,48]. Foremen and project managers had the lowest amount of minutes above 33% RHR during work, the highest amount of minutes above 33% RHR during leisure and were the only professions spending more minutes in high ranges of RHR during leisure than during work. Generally, the present measurements indicated that cardiovascular load in spare-time was low. This may indicate the suggestion that occupations with manual work might have low levels of leisure time physical activity [28]. 

Construction work has been associated to development of MSDs and reduction in work ability, and heavy physical work commonly is considered a major risk factor [1,2]. Our data did not show any significant increase on musculoskeletal complaints or decrease in reported work ability with increasing cardiovascular demands. If musculoskeletal complaints develop over time, and RHR declines with increasing age, this combination could cancel out any possible association. Follow-up investigations of outcome based on these initial objective measurements, may provide more information concerning this issue. Reduction in work ability with high physical demands has also been shown in cross-sectional studies [2]. A recent cross-sectional investigation on cardiovascular load and work ability found that high physical workload was associated with self-reported work ability in women, but not in men [19]. Similarly, Karlqvist *et al.* found that women needing to exceed their physical demands regularly during work had reduced general health and increased level of musculoskeletal complaints, however, men had no such problems [42]. Thus, there may also be sex differences that we did not explore here.

The methods and design used in this study were chosen to provide a thorough objective description of the cardiovascular demands in construction work. The continuous measurement over several days provide a more solid foundation when describing general demands compared to studies with task or short period measurements, which may serve other purposes.

The formula for HR_max_ presented by Tanaka and coworkers [30] produced a standard deviation ~10 bpm for individuals of any age. Hence, the calculation of RHR can only give an approximate measure of cardiovascular load. Since arousal-inducing psychological factors may introduce large errors in measuring HR during rest and moderate levels of physical workload, obtaining valid measurements of HR_min_ is difficult at the workplace. Therefore, we based HR_min_ on the sex- and age-adjusted population means. The study participants were drawn from a variety of occupational titles within construction and will thereby give a good overall description of this work sector. However, they were male employees at Norwegian large-scale enterprises and data may not be generalized small enterprises and builders of private homes. In addition, there is possibility of selection bias of participants. From the 579 invited, 255 answered the questionnaire, 161 volunteered for technical measurement, and a sample of 57 were selected. Still, the participants monitored in the present study did not differ from the questionnaire group in any variable investigated, except smoking and gender. Concerning gender, there were only 18 females in total answering the questionnaire, hence females were weakly represented. However, at present this is the normal gender distribution in the construction sector. Long-term follow-ups are needed to determine the long term health effects of cardiovascular load during work. 

## 5. Conclusions

Cardiovascular demands in construction are characterized by mainly work in ranges of relative heart rate below 39%, with few continuous periods above one-third of capacity. Few minutes are spent in high load intensities needed to achieve training effect. Cardiovascular load differs between professions within construction and both age and aerobic fitness are individual factors influencing cardiovascular load at work.

## Figures and Tables

**Table 1:** Characteristics of the study population divided in technical and questionnaire groups.

Variable	Technical	Questionnaire	Profession	Technical	Questionnaire
	*n* = 42	*n* = 255		*n* = 42	*n* = 255
	*Mean (SD)*	*Mean (SD)*		*Number (%)*	*Number (%)*
Age (years)	40.4 (13.6)	42.7 (12.9)	Project manager	5 (11.9)	52 (20.4)
Male gender (frequency and %) **^a^**	42 (100%) *	237 (93%)	Carpenter	12 (28.6)	70 (27.5)
Height (cm)	179.1 (6.2)	179.6 (7.1)	Bricklayer	5 (11.9)	11 (4.3)
Body mass (kg)	82.5 (11.5)	85.2 (12.9)	Concrete worker	8 (19.0)	41 (16.1)
Body mass index (kg/m^2^)	25.8 (3.5)	26.4 (4.0)	Henchman	7 (16.7)	13 (5.1)
Normal work hours per week	37.7 (4.9)	38.4 (3.7)	Foreman	4 (9.5)	26 (10.2)
Smokers (frequency and %) **^a^**	13 (31%) *	46 (18%)	Working with various tasks	1 (2.4)	16 (6.3)
Perceived health (1–5)	2.6 (0.9)	2.6 (0.9)	Driver	0 (0)	9 (3.5)
Waist circumference (cm)	93.1 (10.4)	NA	Missing	0 (0)	17 (6.7)
HR_max_ (bpm)	179.7 (9.5)	NA	Total	42 (100)	255 (100)
HR_min_ (bpm)	68.7 (0.5)	NA			
Estimated $\dot{V}$O_2max_ (L·min^−1^)	3.1 (0.9)	NA			
Estimated $\dot{V}$O_2max_ (mL·kg^−1^·min^−1^)	38 .4 (10.7)	NA			
Handstrength (kg)	54.6 (8.9)	NA			
Blood pressure systolic (mmHg)	135.2 (12.1)	NA			
Blood pressure diastolic (mmHg)	78.9 (9.2)	NA			

* Significant differences between groups (*p* < 0.05); **^a^** Variable is presented as frequency and percentage.

**Table 2:** Participants experiencing continuous episodes of relative heart rate above 33% during work.

	Continuously ≥ 5 min	Continuously ≥ 10 min	Continuously ≥ 15 min
**Mean number of episodes**			
>0 times	7 persons (16.7%)	5 persons (11.9%)	1 person (2.4%)
≥ 3 times	3 persons (7.1%)	0 persons (0%)	0 persons (0%)
≥ 5 times	2 persons (4.8%)	0 persons (0%)	0 persons (0%)

Mean episodes is an average from the continuous workdays measured. Percentages represented is related to the total measured sample, *n* = 42.

**Table 3:** Unadjusted and adjusted regression analyses with mean percentage relative heart rate at work as depentent variable.

Variable	Unadjusted		Adjusted ^a^	
	Beta	*p*-Value	Beta	*p*-Value
Age (years)	−0.298	**0.021**	−0.414	**0.002**
Body mass index (kg/m^2^)	−0.266	0.613	Not included	Not included
Seniority (years) **^b^**	−0.234	0.085	Not included	Not included
Smoking	1.390	0.377	Not included	Not included
Estimated $\dot{V}$O_2max_ (L·min^−1^)	−5.924	**0.002**	−5.098	**0.008**
Physical activity (1–4)	−3.205	0.109	−2.025	0.304
Hand strength (kg)	0.169	0.401	Not included	Not included
Work ability	−1.844	0.076	−0.255	0.800
Musculoskeletal pain	0.569	0.617	Not included	Not included
Perceived exhaustion at work	1.598	**0.043**	0.713	0.288
Perceived health	1.802	0.346	Not included	Not included

**^a^** Multiple regression including the variables: Age, Estimated $\dot{V}$O_2max_, Physical activity, Work ability and Perceived exhaustion at work. Due to variable to participant ratio, BMI, smoking, hand strength, musculoskeletal complaint-severity index and perceived health were excluded from the adjusted model because of low explainatory value; **^b^** seniority was not included in the adjusted model due to high (r = 0.845) significant (*p* < 0.001) correlation to age; Model r^2^ = 0.454.
